# Spatial distribution and reduction of PCDD/PCDF Toxic Equivalents along three shallow lowland reservoirs

**DOI:** 10.1007/s11356-013-2401-7

**Published:** 2013-12-12

**Authors:** M. Urbaniak, E. Kiedrzyńska, M. Zieliński, W. Tołoczko, M. Zalewski

**Affiliations:** 1 European Regional Centre for Ecohydrology, Polish Academy of Sciences, Tylna 3, 90-364 Lodz, Poland; 2 Department of Applied Ecology, Faculty of Biology and Environmental Protection, University of Lodz, Banacha 12/16, 90-237 Lodz, Poland; 3 Nofer Institute of Occupational Medicine, Teresy 8, 91-348 Lodz, Poland; 4 Department of Physical Geography, Faculty of Geography, University of Lodz, Narutowicza 88, 90-139 Lodz, Poland

**Keywords:** PCDDs/PCDFs, Sediments, Reservoir, Toxicity reduction

## Abstract

Reservoirs situated along a river continuum are ecosystems where rates of transfer of suspended matter and associated micropollutants are reduced due to sedimentation, accumulation, and biological and physical transformation processes. Among the micropollutants, PCDDs and PCDFs are substances that are highly toxic and carcinogenic for humans and animals. They are emitted and dispersed in the environment throughout the whole catchment area and may accumulate in aquatic and terrestrial food chains, creating a risk for human health. A wealth of data exists indicating the increase in the concentrations of pollutants along a river continuum. A comparative analysis of total, individual, and TEQ PCDD/PCDF concentrations in large lowland, shallow reservoirs located in different catchments (“I”—industrial/urban/agricultural, “U”—urban/agricultural, and “A”—agricultural/rural) showed decreases of the TEQ concentrations in bottom sediments along a gradient from the middle sections to the dam walls. Moreover, penta-, hexa-, and heptachlorinated CDD/CDF congeners were reduced from 28.8 up to 93.6 % in all three types of reservoirs. A further analysis of water samples from the inlets and outlets of the “A” reservoir confirmed this tendency.

## Introduction

A river catchment is a highly complex ecological system wherein hydrological pulses facilitate a transfer of nutrients, mineral, and organic matter along the river continuum (Vannote et al. 1980; Bowes et al. 2003). Various forms of human activity in a river basin result in a tendency to increase the amounts of emitted pollutants, such as PCDDs and PCDFs (Klanowa et al. 2007; Lohman et al. 2007; Nieuwoudt et al. 2009; Urbaniak et al. 2013).

The plethora of data available on the transfer of PCDDs/PCDFs along a river continuum suggests that their concentration undergoes a concomitant permanent downstream increase (Camusso et al. 2000; Kannan et al. 2001; Kowalewska et al. 2003; Hilscherova et al. 2003; Koh et al. 2004; Konieczka et al. 2005; Urbaniak et al. 2010b; Niemirycz and Jankowska 2011; Urbaniak et al. 2012a, b, 2013).

Reservoirs constructed usually in the middle or downstream section of the river, intersect with its continuum and, thus, affect the pollutant transportation processes. The traditional view is that large reservoirs are considered to have negative effects on the structure and functionality of river ecosystems (Hu et al. 2008; Zhai et al. 2010). One of the main negative impacts of large, traditionally constructed dams on the environment is degradation of the continuity of the river, which reduces the migration of aquatic organisms along it (Ward and Stanford 1983; Miller 2005). However, in the face of global climate and anthropogenic changes, large reservoirs play also an increasingly important role in compensating for those changes by enhancing the available water resources and contributing to human development by acting as reliable sources of drinking and irrigation water, hydropower, food, recreation, navigation, income, and other important benefits (WCD World Commission on Dams 2000).

Reservoirs also play a role as traps for suspended matter (Krasa et al. 2005; Kentzer et al. 2010; Li et al. 2011; Ran et al. 2013) and, consequently, for associated chemical toxicants (Devault et al. 2009; Chi et al. 2011), thus improving the water quality in downstream and coastal zones (Kentzer et al. 2010). A decrease in flow velocity and an increase of flocculent settling in reservoirs create excellent conditions for the sedimentation and deposition of pollutants. Thus, reservoirs can act as sinks for contaminants and, therefore, are important for pollution studies and monitoring of ecosystem stress (Chi et al. 2007, 2011). Our earlier study (Urbaniak et al. 2012a) demonstrated a decrease in the concentration of total dl-PCBs non-ortho PCBs, and mono-ortho PCBs along the lowland reservoir sediments of 29, 45, and 25 %, respectively. Moreover, the results of the sediment samples collected from the river above and below the studied reservoir showed a 79 % reduction of the total dl-PCBs content and TEQ concentration below the dam. This clearly indicates the potential for a lowland reservoir in the reduction of pollutant concentrations transported along the river continuum. Consequently, as suggested by Zalewski (2010), in a highly modified catchment (agricultural/urban), where the river continuum and flood processes are highly degraded, the benefits of dams may seriously outweigh their negative impact. Assuming this to be the case, the hypothesis that reservoirs may reduce the toxicity of PCDDs/PCDFs transferred downstream was tested by conducting a comparative analysis of the concentrations and toxicity of PCDDs/PCDFs transported along the three shallow lowland reservoirs. This hypothesis was further validated by analysis of PCDDs/PCDFs, suspended particulate matter, particulate inorganic matter, particulate organic matter, and phosphorus concentration in water inflow and outflow from the “A” reservoir.

## Material and methods

### Study area

**Fig. 1:**
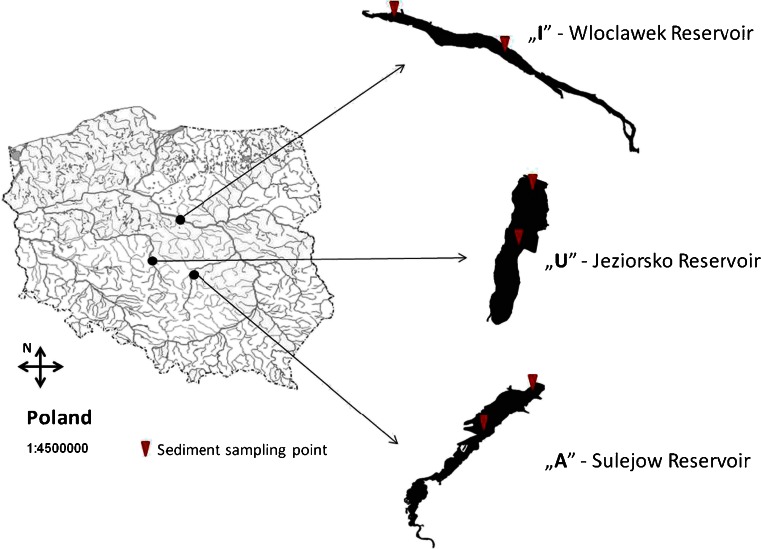
Study area. “*I*” Industrial/urban/agricultural, “*U*” urban/industrial, “*A*” agricultural/rural

The study area includes three types of reservoirs:

- “I”—Wloclawek Reservoir (Vistula River basin)—a reservoir situated in an industrial/urban/agricultural catchment. The reservoir was constructed in 1970 as the largest man-made lake in Poland. The reservoir is situated in the middle course of the Vistula River, 670 km along the length of the river (Fig. 1). Its surface covers 75 km^2^, with a maximum capacity of 408 M m^3^. The length is 58.0 km, width 2.40 km, average depth 5.50 m, and average retention time about 5–3 days. Nevertheless, the reservoir has a riverine character, demonstrated by the water retention time falling below 1 day during high water levels. During the research period of 2007 and 2008, an average annual river flow of 930–970 m^3^ s^−1^ and high rate of sediment accumulation caused the character of the reservoir to become more riverine, resulting in a lack of water retention. In total, 65.0 % of the Vistula River catchment above the reservoir is covered by arable lands (16 % being meadows and grasslands), 27.0 % by forests cover, and 7.30 % by urbanized/industrial areas (Bielecka and Ciołkosz 2004; Grzesiak and Domańska 2006; Tamm et al. 2009) (Table 1).
- “U”—Jeziorsko Reservoir (Warta River basin)—a reservoir constructed in 1986, situated in an urban/agricultural catchment. It is located in the middle course of the Warta River, the largest tributary of the Oder River (Fig. 1). At its maximum capacity, the reservoir covers 42,300 m^2^ with a total capacity of 203 M m^3^ and average depth of 4.10 m. At the catchment area in agricultural land, a dense network of mid-sized and small towns and villages dominates. Arable lands cover approximately 60.0 % of the river catchment above the reservoir (including 14 % covered by meadows and pastures). Forests cover 25.3 %, and 14.7 % is covered by urbanized/industrial areas (Szyper and Mastyński 1997; Galicka et al. 2007; Kuligowski 2007) (Table 1).
- “A”—Sulejow Reservoir (Pilica River basin)—a reservoir constructed in 1973, situated in an agricultural/rural catchment, located in the middle course of the Pilica River (Fig. 1). Its maximum length is 15.5 km, and the maximum width is 2.10 km. At maximum capacity (75 M m^3^), the reservoir covers 22.0 km^2^, with a mean depth of 3.30 m, and a maximum depth of 11.0 m. Arable lands cover 64 % of the river catchment above the reservoir, forests 27.0 % and urbanized/industrial areas 9.00 % (Ambrożewski 1993 , 1996; Galicka 1996) (Table 1).

**Table 1:** Characteristics of the studied “I”, “U”, and “A” reservoirs

Reservoir type	“I”	“U”	“A”
River	Vistula	Warta	Pilica
Average flow *Q* (m^3^ s^−1^)	561 (Warsaw)	127	21.2
Max. flow *Q*_max_ (m^3^ s^−1^)	5,860	972	139
Min. flow *Q*_min_ (m^3^ s^−1^)	110	21.2	n.d.
Length (km)	1,070	808	319
River catchment above the reservoir			
Surface (km^2^)	171,000	9,190	4,880
Cities (above 100,000 inhabitants)	15	2	0
Forests (%)	27 (46,200 km^2^)	25.3 (2,320 km^2^)	27 (1,210 km^2^)
Agricultural area (%)	65 (111,150 km^2^)	60 (5,517 km^2^)	64 (2,870 km^2^)
Urbanized/industrial area (%)	7.3 (12,500 km^2^)	14.7 (1,350 km^2^)	9 (440 km^2^)
Direct catchment of the reservoir			
Surface (km^2^)	171	538	4,880
Forests (%)	61.5	16.4	26.9
Agricultural land (%)	n.d.	71.1	64.1
Reservoir			
Year of construction	1970	1986	1973
Max. surface (m^2^)	75 M	42.3 M	23.8 M
Min. surface (m^2^)	n.d.	17.6 M	6.3 M
Max. depth (m)	14.0	4.80	11.0 m
Average depth (m)	5.50	4.10	3.30 m
Capacity (m^3^)	370–408 M	203 M	70.0 M
Average width (km)	2.40	n.d.	1.50
Max. width (km)	3.30	3.50	2.00
Length (km)	55.0	16.3	17.0
Average flow (m^3^ s^−1^)	930–970	49.0	27.0
Average retention time (days)	3.00–5.00	21.5	42.0

*n.d*. Data not available, “*I*” industrial/urban/agricultural, “*U*” urban/industrial, “*A*” agricultural/rural

### Sediments

#### Sediment sampling for PCDDs/PCDFs

Sediment samples (10–25 cm thickness) were collected from the middle and dam section of each reservoir (Fig. 1) four times during the research time: on April 2007 and 2008 and on October 2007 and 2008. The sediment samples from each reservoir section were collected in triplicate and were freeze-dried (−40 °C, 1 mba, 72 h; Edwards Freeze Dryer), sieved through a 2-mm mesh sieve and mixed in proportion 1:1:1 in order to obtain one representative sample for the studied middle and dam section of each reservoir (Urbaniak et al. 2008, 2009a, b, 2010a, b, 2012a, 2013).

#### Analysis of PCDDs/PCDFs in sediments

All analytical work has been performed in the accredited Laboratory of the Environmental Organic Pollutants Monitoring at the Nofer Institute of Occupational Medicine, Lodz, Poland. The analytical laboratory involved in 2005 and 2009 successfully passed the accreditation procedure.

The pretreatment of the sediment samples were performed according to PN-EN 1948-3 (2002) and EPA Method 1613. Two grams of each sediment sample was spiked with isotopically labeled standards (Cambridge Isotopes Laboratories, USA) and extracted by Accelerated Solvent Extraction 200 Dionex at 150 atm (11 MPa), and the oven was heated to 175 °C with toluene. The extracts were purified with multilayer silica columns packed with neutral, acidic, and basic silica gel and eluted with 200 mL of hexane. The hexane extracts were further concentrated to 5.00 mL by rotary evaporation and concentrated to 100 μL under a gentle stream of nitrogen, replacing the *n*-hexane with nonane.

The identification and quantification of seven congeners of PCDDs and 10 congeners of PCDFs, identified by the WHO as potentially toxic, were performed by high-resolution gas chromatography (HRGC)/high-resolution mass spectrometry (HRMS): an HP 6890 N Agilent Technologies GC coupled with a high resolution mass spectrometer (AutoSpec Ultima). The GC was operated in the splitless injection mode and, for the HRMS, perfluorokerosene was used as a calibration reference (lock mass).

The oven temperature protocol was 150 °C for 2 min, 20 °C min^−1^ to 200 °C (0 min), 1 °C min^−1^ to 220 °C for 16 min and 3 °C min^−1^ to 320 °C for 3 min. The injector temperature was 270 °C. The mass spectrometer was operated under positive electron ionization conditions: 34.8 eV electron energy at a resolving power of 10,000 with an ion source temperature of 250 °C. Helium was used as a carrier gas at a flow rate of 1.60 mL min^−1^. Samples were quantified with the isotope dilution method.

### Water

#### Water sampling for PCDDs/PCDFs

Water samples from the Sulejow Reservoir's “A” inflow and outflow (Fig. 1) were collected twice—once during the high (spring) and once during the serene (summer) water stages in 2010 using 5.00-L Teflon jars. After collection, samples were directly transported to the laboratory, where they were extracted and purified for PCDDs/PCDFs.

#### Analysis of PCDDs/PCDFs in water

All analytical work has been performed in the accredited Laboratory for Trace Organic Analyses at the Cracow University of Technology, Cracow, Poland. The laboratory is involved in the Circuit INterlaboratories for Dioxins organized by the Interuniversity Consortium, “Chemistry for the Environment”, in collaboration with LabService Analytica S.r.l.

The applied analytical methods were properly validated and the laboratory successfully passed the accreditation procedure. The accreditation granted by the Polish Centre for Accreditation No. AB 749 is valid till August 2014.

Water samples of 2.00 L of were spiked with 60.0 pg of 17 of ^13^C-labeled PCDDs/PCDFs (NK-LCS-G and WP-LCS, respectively, obtained from Wellington Laboratories), and liquid/liquid extracted with toluene. Toluene extract after rotary evaporation to ca. 20.0 mL was cleaned up as follows: concentrated extract was placed in the bottom sealed polyethylene semipermeable membrane tube of 80.0 μm wall thickness and cleaned up overnight with 100 mL hexane (the outer solvent). The hexane dialysate was cleaned up on a silica gel column coated with 44.0 % sulfuric acid and alumina according to EPA 1613 method. The final extract was spiked with 20.0 μL of precision and recovery solution (EPA 1613 ISS mix of 200 ng/mL of ^13^C_12_-1,2,3,4-TCDD and ^13^C_12_-1,2,3,7,8,9-HxCDD) prepared in nonane and evaporated to 20.0 μL in a gentle stream of nitrogen.

Determination of 17 PCDDs/PCDFs was performed by isotope dilution gas chromatography–mass spectrometry (ID-GC/MS–MS) on a Thermo Scientific GCQ-1100/Trace2000 system equipped with Xcalibur data acquisition and analysis software. Separation was performed on a 30.0 m × 0.250 mm i.d. DB5MS J&W capillary column of 25-μm film and DB17 30.0 m × 0.250 mm i.d. A sample of 2.50 μL volume was injected into a SSL injector at 260 °C. The GC oven was programmed as follows: an initial temperature of 130 °C held for 3 minutes, the temperature ramp of 50 °C/minute to 180 °C and then another temperature ramp 2 °C/minute to 270 °C. Finally, the temperature ramp was 20 °C/minute to 300 °C and held for 5 minutes. The result uncertainty was expressed as extended measurement uncertainty for *k* = 2 at the confidence level of 95 %.

#### Water sampling for SPM, phosphorus concentrations, and loads

Additionally, in 2010, water samples for analysis of suspended particulate matter (SPM), particulate inorganic matter (PIM), particulate organic matter (POM), soluble reactive phosphorus (SRP), and total phosphorus (TP) concentrations in the Sulejow Reservoir, “A”, were collected every 4 days at two stations: (1) inflow to the reservoir (135 water samples) and (2) outflow from the reservoir (98 water samples).

#### Analysis of SPM, phosphorus concentrations, and loads

The concentrations of SPM in the water were measured with a PIHM bathometer. Water samples were immediately filtered through Whatman GF/F (0.450 μm) filters. The SPM was estimated by drying the Whatman GF/F filters at 105 °C and weighing them on a laboratory scale. The PIM was estimated by loss on ignition at 550 °C (Ostrowska et al. 1991). The POM was estimated as the difference between SPM and PIM. Filtered water samples for analyses of SRP were measured with an ion chromatography system (DIONEX, ICS 1000). TP was analyzed in unfiltered water with the addition of Merck Oxisolv reagent using the Merck MV 500 Microwave Digestion System and determined by the ascorbic acid method (Greenberg et al. 1992). The SPM, PIM, POM and, SRP and TP load in the inflow and outflow from the Sulejow Reservoir was calculated with daily discharges of the Pilica River.

### Statistical analyses

All results were subjected to statistical analyses using Statistica software for Windows. The Wilcoxon tests were used to compare the concentrations, or amounts, of substances between the two sampling points within a single reservoir. The statements of significance were based on a probability level of *P* ≤ 0.05.

## Results

To assess the spatial distribution of PCDDs/PCDFs and the reduction of their toxicity along large lowland reservoirs, the sediment samples from the middle and dam sections of the three reservoirs located in different catchment types, “I”—industrial/urban/agricultural, “U”—urban/agricultural, and “A”—agricultural/rural, were collected and analyzed for the individual, total, and TEQ concentrations of PCDDs/PCDFs, as were the water samples from the inlet and outlet of the reservoir. The water samples were further analyzed with regard to SPM, phosphorus concentration, and load. The obtained results are described below.

### Spatial differences of PCDDs/PCDFs in the sediments of the “I” reservoir

The total concentration of PCDDs/PCDFs in the sediments from the “I” reservoir ranged from 765 to 515 ng kg^−1^, for the middle and dam sampling sites, respectively (Table 2). The obtained concentrations were significantly higher at the middle sampling point, according to the Wilcoxon test. Similarly, the sum of PCDDs and the sum of PCDFs were significantly higher (Wilcoxon test) in the middle section of the reservoir and ranged from 676 to 88.6 ng kg^−1^ in the middle to 445 and 70.7 ng kg^−1^ in the dam section for PCDDs and PCDFs, respectively (Tables 2 and 3).

**Table 2:** The spatial changes in PCDD/PCDF concentrations and organic matter content along the studied “I”, “U”, and “A” reservoirs

Reservoir type	“I”		“U”		“A”	
Site	Middle	Dam	Middle	Dam	Middle	Dam
2378-TCDD	0.0700 ± 1.25	0.670 ± 0.140	n.d.	n.d.	0.0200 ± 0.0300	n.d.
12378-PeCDD	3.20 ± 1.70	0.850 ± 6.39	3.68 ± 3.88	0.600 ± 0.740	0.0600 ± 0.110	0.0600 ± 0.130
123478-HxCDD	3.43 ± 2.65	2.56 ± 6.60	3.63 ± 4.69	0.960 ± 1.92	0.290 ± 0.340	0.220 ± 0.440
123678-HxCDD	5.73 ± 5.51	5.04 ± 8.88	3.88 ± 7.22	2.11 ± 3.04	0.850 ± 1.540	n.d.
123789-HxCDD	8.74 ± 5.14	4.00 ± 14.3	5.72 ± 9.80	2.72 ± 3.47	0.990 ± 1.170	1.27 ± 1.75
1234678-HpCDD	47.6 ± 51.3	32.0 ± 53.4	20.6 ± 10.6	36.9 ± 8.92	9.88 ± 9.76	8.32 ± 6.56
OCDD	607 ± 681	399 ± 625	194 ± 113	446 ± 191	141 ± 145	201 ± 181
Sum of PCDDs (ng kg^−1^ d.w.)	**676** ± **742**	**445** ± **713**	**232** ± **128**	**489** ± **204**	**154** ± **155**	**211** ± **189**
2378-TCDF	2.52 ± 2.42	1.76 ± 3.52	0.510 ± 1.02	1.31 ± 2.27	0.470 ± 0.510	0.750 ± 0.610
12378-PeCDF	4.71 ± 2.34	1.18 ± 8.35	7.23 ± 7.73	n.d.	0.570 ± 0.770	0.690 ± 0.550
23478-PeCDF	4.48 ± 3.76	3.41 ± 7.52	0.310 ± 0.430	0.110 ± 0.220	0.570 ± 0.500	n.d.
123478-HxCDF	17.9 ± 8.75	20.7 ± 13.6	3.05 ± 4.46	1.65 ± 2.59	1.53 ± 1.75	0.650 ± 0.640
123678-HxCDF	4.20 ± 2.06	2.55 ± 5.16	0.250 ± 0.320	1.33 ± 2.66	1.18 ± 1.52	0.770 ± 1.54
234678-HxCDF	11.2 ± 9.12	12.8 ± 11.1	3.42 ± 5.64	5.76 ± 5.25	2.74 ± 3.86	0.600 ± 0.800
123789-HxCDF	5.39 ± 3.21	2.56 ± 10.1	6.87 ± 13.0	1.67 ± 3.35	0.790 ± 1.58	0.150 ± 0.290
1234678-HpCDF	8.84 ± 8.27	5.50 ± 10.8	5.13 ± 8.06	5.13 ± 5.52	7.18 ± 8.43	3.15 ± 1.02
1234789-HpCDF	5.74 ± 3.17	2.27 ± 7.31	3.96 ± 7.91	2.30 ± 4.61	0.940 ± 1.00	1.29 ± 1.26
OCDF	23.6 ± 21.7	18.0 ± 26.2	7.12 ± 11.8	9.42 ± 4.59	6.36 ± 5.44	5.45 ± 2.49
Sum of PCDFs (ng kg^−1^ d.w.)	**88.6** ± **57.1**	**70.7** ± **100**	**37.8** ± **55.5**	**28.7** ± **26.7**	**22.3** ± **23.0**	**13.5** ± **6.57**
Sum of PCDDs/PCDFs (ng kg^−1^ d.w.)	**765** ± **799**	**515** ± **813**	**269** ± **183**	**518** ± **230**	**176** ± **178**	**224** ± **195**
TEQ (ng TEQ kg^−1^ d.w.)	**11.5** ± **17.0**	**8.30** ± **6.91**	**5.47** ± **8.56**	**2.64** ± **3.04**	**1.38** ± **1.27**	**0.710** ± **0.540**
Organic matter content (%)	**8.80** ± **0.0400**	**9.40** ± **0.0500**	**0.82** ± **0.02**	**1.34** ± **0.0200**	**5.12** ± **0.0700**	**8.00** ± **0.300**

Bold values refer to the sum of PCDD congeners, sum of PCDF congeners, sum of PCDD/PCDF congeners, TEQ concentration and organic matter content

“*I*” Industrial/urban/agricultural, “*U*” urban/industrial, “*A*” agricultural/rural

**Table 3:** The results of statistical analysis using the Wilcoxon matched pair test

Compared congeners	Reservoir type		
	“I” M vs. D	“U” M vs. D	“A” M vs. D
PCDDs	*P* = 0.03	*P* = 0.50	*P* = 0.75
PCDFs	*P* = 0.05	*P* = 0.92	*P* = 0.08
PCDDs/PCDFs	*P* = 0.004	*P* = 0.57	*P* = 0.03
TEQ	*P* = 0.68	*P* = 0.15	*P* = 0.04

In all of the analyzed samples, the percentage content of PCDDs was higher than PCDFs, the PCDDs constituting 79.1 % of the total PCDDs/PCDFs content. The reason for this may be associated with the great amount of OCDD, ranging from 16.0 to 86.4 % of the total content of PCDDs/PCDFs and from 47.7 to 94.4 % of the total content of seven PCDD congeners. A higher percentage content of PCDDs, 88.4 %, was also noted at the middle site, whereas at the dam section, this value was 79.9 %.

Changes in the PCDD profiles were also observed along the examined sampling sites with the percentage content of five PCDD congeners increasing from 1.11 to 5.99 % at the dam location. The exceptions were congeners 1,2,3,7,8-PeCDD and OCDD, whose content decreased by 0.130 and 13.1 %, respectively. The reverse situation was observed for the PCDF profiles with seven congeners decreasing in content and three congeners increasing at the dam site. The higher TEQ concentrations of the analyzed samples were identified at the middle site (11.5 ng TEQ kg^−1^) in comparison to these noted at the dam site (8.30 ng TEQ kg^−1^). The obtained values were generated by the high content of the PCDD congeners, especially 1,2,3,7,8-PeCDD, which possesses a high TEF value (Table 2).

### Spatial differences of PCDDs/PCDFs in the sediments of the “U” reservoir

The total concentration of 17 PCDD/PCDF congeners in the sediments of “U” reservoir ranged from 269 ng kg^−1^ at the middle to 518 ng kg^−1^ at the dam site. The Wilcoxon test showed no significant differences between the above concentrations (Table 3). A similar situation was observed for the sum of seven PCDD congeners, where the average concentration at the middle site was 232 and 489 ng kg^−1^ at the dam site (Table 2). No significant differences between the PCDD concentrations at these sites were identified by the Wilcoxon test (Table 3).

The total concentration of the 10 PCDF congeners was several times lower and amounted for 37.9 ng kg^−1^ at the middle and 28.7 ng kg^−1^ at the dam sections, and unlike the PCDDs content, was higher at the middle part of the reservoir (Table 2). Nevertheless, the Wilcoxon test showed no significant differences between the examined middle and dam sites (Table 3).

The most frequently observed congeners in all the analyzed samples were PCDDs, comprising up to 91.9 % of the total PCDDs/PCDFs content (88.8 and 95.0 % at the middle and dam sections, respectively), with the OCDD congener predominating. The total PCDFs contribution accounted for 11.2 and 5.09 %, at the middle and dam sites, respectively, with an average value of 8.14 %.

Differences regarding individual PCDD congener profiles were also observed along the sampling sites: in particular, the OCDD congener concentration was seen to increase by 7.00 % between the middle and dam sites. PCDF profiles also revealed that the percentage contributions of four congeners increased: 2,3,7,8-TCDF by 11.6 %, 123678-HxCDF by 2.32 %, 2,3,4,6,7,8-HxCDF by 13.0 %, and OCDF by 19.6 %. The content of the other six PCDF congeners decreased: from 0.0900 % for 1,2,3,4,7,8,9-HpCDF to 30.0 % for the highly toxic 1,2,3,7,8-PeCDF.

Regarding the toxicity level of the analyzed samples, in contrast to the total concentrations, the TEQ values were found to be lower at the dam (2.64 ng TEQ kg^−1^) than the middle part of the reservoir (5.47 ng TEQ kg^−1^) (Table 2). Nevertheless, according to the Wilcoxon test, no significant differences existed between them (Table 3).

### Spatial differences of PCDDs/PCDFs in the sediments of the “A” reservoir

The concentrations of total PCDDs/PCDFs in sediments taken from the “A” reservoir ranged from 176 to 224 ng kg^−1^ at the middle and dam sections, respectively. Similarly, the PCDD concentrations showed higher values at the dam site: 211 ng kg^−1^ compared to 154 ng kg^−1^ at the middle position. However, the PCDF concentrations were higher at the middle section than at the dam site: 22.3 and 13.5 ng kg^−1^, respectively (Table 2). While the statistical analysis revealed no significant differences in the mean concentrations of PCDD and mean concentrations of PCDF between the two positions, differences were found in the average concentrations of PCDD/PCDF (Table 3).

In all the samples, PCDDs predominated, compared to PCDFs, with a total average percentage value of 85.3 %, 89.9 % at the middle position and 84.4 % at the dam site. The percentage of PCDFs ranged between 1.07 and 36.1 %: an average of 14.7 %. Also, the dominant congener of this reservoir was OCDD, which helped generate the high PCDD/PCDF ratio. The percentage content of OCDD at the middle site was 6.25 % higher than the dam position. In the case of the hexa-CDD and penta-CDD congeners, a small decline in its percentage content not exceeding 1.00 % was observed. The PCDF profiles were characterized by a higher content of less chlorinated congeners, such as 2,3,4,7,8-PeCDF, 2,3,4,6,7,8-HxCDF, and 1,2,3,4,6,7 0.8-HpCDF. Regarding the toxicity level of the analyzed samples, a significantly higher value was recorded in the samples collected at the middle position (1.38 ng TEQ kg^−1^) compared to the dam part of the reservoir, wherein the TEQ concentration was half that of the middle position (0.710 ng TEQ kg^−1^) (Tables 2 and 3).

### A reduction of PCDDs/PCDFs along the studied reservoir sediments

It was found that the studied reservoirs “I”, “U”, and “A”, were heterogeneous with regard to total PCDD/PCDF concentrations. In the “U” and “A” reservoirs, the higher total concentration of PCDDs/PCDFs was detected at the dam part. This tendency was not observed in case of “I” reservoir, wherein a decrease in the total PCDDs/PCDFs concentration toward the dam wall was observed (Table 2). In contrast, the TEQ concentrations showed a decrease along all three studied reservoirs (Table 2) with a significant reduction noted for the “A” reservoir, according to the Wilcoxon test (Table 3).

The analysis showed also a significant decrease in the concentrations of the five homologue groups of PCDD/PCDF congeners (tetra-, penta-, hexa- hepta-, and octa-CDD/CDF) along the reservoirs. The greatest reductions were observed for penta-CDD/CDF, ranging from 38 to 94 %, and hexa-CDD/CDF congeners, ranging from 11.3 to 56.1 %, whereas tetra- and octa-CDD/CDF demonstrated the lowest decreases in “I” reservoir, with 6.17 %, and 33.9 %, respectively, and increased concentrations in the “U” and “A” reservoirs (Table 4).

**Table 4:** Summary of PCDD/PCDF reduction along the along the studied “I”, “U”, and “A” reservoirs

Group of compounds	Reservoir type								
	“I”			“U”			“A”		
	M (ng kg^-1^)	D (ng kg^−1^)	Reduction (%)	M (ng kg^−1^)	D (ng kg^−1^)	Reduction (%)	M (ng kg^−1^)	D (ng kg^−1^)	Reduction (%)
Tetra-CDD/CDF	2.59	2.43	**6.17**	0.510	1.31	−*157*	0.490	0.750	−*53.1*
Penta-CDD/CDF	12.4	5.44	**56.1**	11.2	0.710	**93.6**	1.20	0.750	**37.5**
Hexa-CDD/CDF	56.6	50.2	**11.3**	26.8	16.2	**39.5**	8.37	3.66	**56.3**
Hepta-CDD/CDF	62.1	39.8	**35.9**	29.7	44.4	−*49.5*	18.0	12.8	**28.8**
Octa-CDD/CDF	631	417	**33.9**	201	456	−*126*	148	206	−*39.2*

The bold values refer to the reduction percentage calculating on the basis of the not-bolded values. The italic values refer to the obtained negative values of reduction percentage

“*I*” Industrial/urban/agricultural, “*U*” urban/industrial, “*A*” agricultural/rural

### A reduction of PCDDs/PCDFs, SPM, and phosphorus in the reservoir outflow water

**Fig. 2:**
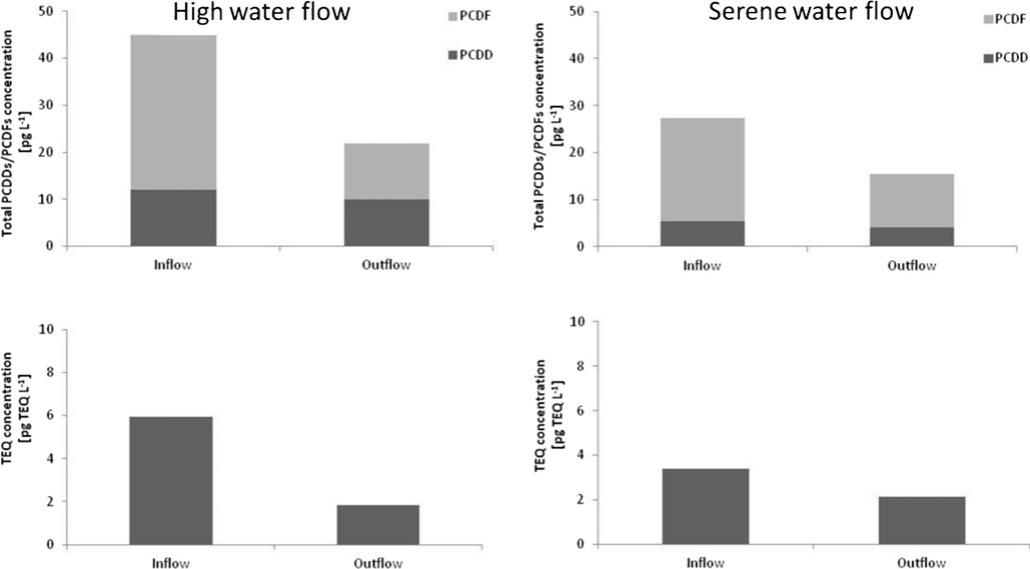
A reduction of the total PCDD/PCDF concentrations (expressed as the sum of the analyzed PCDD and PCDF) and TEQ concentrations by the “A” reservoir during high and serene water flow

The above observation was supported by further results of water samples collected from the inflow and outflow of the “A” reservoir. The obtained results demonstrated a significant decrease in the total amount of PCDDs and PCDFs as well as TEQ concentrations (Fig. 2). A decrease of about 21 % of the total PCDDs/PCDFs content (from 111 to 87.5 pg L^−1^) during the high water level and 24.0 % (from 51.1 to 38.7 pg L^−1^) during the serene water level was observed (Fig. 2). The highest decreases were noted for PCDFs, the percentages being 64.0 and 48.0 % for samples collected at the high and serene water stages, respectively. The reductions noted for total PCDDs content were smaller and amounted to 17.0 and 23.0 % during the high and serene water levels, respectively (Fig. 2).

Regarding the PCDD and PCDF homologue groups, the greatest reductions were noted for hexa- (89.1 %) and tetra-CDD/CDF (70.2 %) during high and serene water levels, respectively (Table 5). The lowest decreases, in turn, were noted for the higher chlorinated congeners: 69.0 % for octa-CDD/CDF at the high water stage and 28.6 % for hepta-CDDs/CDFs at the serene water stage (Table 5). The TEQ concentration in the reservoir was also found to be reduced from 5.90 to 1.80 pg TEQ L^−1^ (a reduction of about 69.0 %) during high flows and from 3.40 to 2.10 pg TEQ L^−1^ (a reduction of about 36.0 %) during the serene flow period (Fig. 2).

**Table 5:** Reduction of PCDD/PCDF homologue concentrations by the “A” reservoir

Group of compounds	High water level			Low water level		
	Inflow (pg L^−1^)	Outflow (pg L^−1^)	Reduction (%)	Inflow (pg L^−1^)	Outflow (pg L^−1^)	Reduction (%)
Tetra-CDD/CDF	3.08	0.86	**72.1**	3.02	0.90	**70.2**
Penta-CDD/CDF	6.73	0.91	**86.5**	1.95	1.30	**33.3**
Hexa-CDD/CDF	19.5	2.12	**89.1**	4.27	1.70	**60.2**
Hepta-CDD/CDF	10.5	1.32	**87.4**	2.24	1.60	**28.6**
Octa-CDD/CDF	5.10	1.58	**69.0**	2.79	1.50	**46.2**

The bold values refer to the reduction percentage calculating on the basis of the not-bolded values

The obtained results showed an 81.3 % reduction of the mean annual concentrations of SPM from reservoir inflow (39.7 mg L^−1^) to outflow (7.42 mg L^−1^) (Table 6). Furthermore, 28.2 and 14.6 % decreases in mean TP and SRP concentrations were observed in the Sulejow Reservoir: inflow 209 μg TP L^−1^, outflow 150 μg TP L^−1^ for TP, and inflow 77.2 μg SRP L^−1^, outflow 65.9 μg SRP L^−1^ for SRP (Table 6).

**Table 6:** Mean concentrations of suspended particulate matter (SPM), particulate inorganic matter (PIM), particulate organic matter (POM), and soluble reactive phosphorus (SRP) and total phosphorus (TP) in water inflow and outflow from the “A” reservoir

Concentrations	TP (μg L^−1^)	SRP (μg L^−1^)	PIM (mg L^−1^)	POM (mg L^−1^)	SPM (mg L^−1^)
Inflow to the reservoir with the Pilica River	209	77.2	26.8	12.9	39.7
Outflow from the reservoir	150	65.9	4.35	3.08	7.42
Reduction of concentrations in the reservoir	**59.3**	**11.3**	**22.5**	**9.83**	**32.3**
% Reduction	**28.2**	**14.6**	**83.8**	**76.1**	**81.3**

Bold values refer to the reduction of concentrations in the reservoir and % reductions calculated on the basis of inflow and outflow concentrations of TP, SRP, PIM, POM and SPM

The total river inflow into the Sulejow Reservoir amounted to 1,660 M m^3^, divided between the Pilica (1,410 M m^3^) and Luciaza rivers (258 M m^3^) (Table 7). The total outflow from the dam of the reservoir was 1,330 M m^3^, and evapotranspiration and filtration together amounted to 50.7 M m^3^ (Table 7). The remaining water volume was retained in the reservoir. The results also show a significant retention of SPM and phosphorus load in the reservoir, which amounts to 78.0 % of the SPM (36,086.9 tons), 38.0 % of the TP (120 tons), and 20.0 % of the SRP (23.1 tons) load which flowed into the reservoir in the hydrological year 2010 (Table 7).

**Table 7:** Transport of the SPM, PIM, POM, and TP and SRP load to and from the “A” reservoir and its retention in the “A” reservoir in the hydrological year 2010

Loads	Flow (mln m^3^)	TP (ton)	PO4 (ton)	PIM (ton)	POM (ton)	SPM (ton)
Total river inflow to the reservoir	1,660					
—Inflow with the Pilica River	1,410	312	114	33,700	12,400	46,200
—Inflow with the Luciaza River	258					
Outflow from the reservoir	1,330	193	90.5	6,000	4,060	10,100
Evapotranspiration and filtration	50.7					
Retention in the reservoir	**283.3**	**120**	**23.1**	**27**,**700**	**8**,**350**	**36**,**100**
Retention in the reservoir (%)	**20.0**	**38.0**	**20.0**	**82.0**	**67.0**	**78.0**

Bold values refer to the retention of water, TP, PO4, PIM, POM and SPM and % retention of water, TP, PO4, PIM, POM and SPM in the reservoir calculated on the basis of inflow and outflow loads of above parameters

## Discussion

### Changes in sediment contamination along the three large reservoirs

Taking into account the reservoir and catchment size and the catchment impact of the studied reservoirs (Table 1), the findings show that the highest potential for PCDD/PCDF accumulation exists in the largest and oldest reservoir situated in the highly industrialized/urbanized/agricultural (“I”) catchment, with a high load of industrial effluent (Table 2). This phenomenon may be connected with the association of PCDDs/PCDFs with suspended matter in industrial effluents as well as sewage from residential and commercial land use. Moreover, because of the river character of the “I” reservoir, the highest increase in the rate of sedimentation is in its widest section, the middle, and there is a consequent higher deposition of PCDDs/PCDFs in this part.

The contamination of sediment from the “A” reservoir, located on the Pilica River in the agricultural and forestry basin, was considerably lower compared to that of the “I” reservoir discussed above. This may indicate the decisive role played by the catchment land use of the river above the reservoir, characterized by a lack of large industrial and urban centers.

The concentrations of PCDDs/PCDFs in the sediments of the “U” reservoir were twice those in the sediments of the above described “A” reservoir. This may be explained by the location of two urban centers with a population of above 100,000 inhabitants (Table 1) in the catchment above the “U” reservoir, which may cause greater pollution of the water in the Warta River. The spatial distribution of total PCDD/PCDF content in the ‘U” reservoir showed an increase in the total concentration along the reservoir from its middle to the dam section. This may be a result of the presence of existing fish ponds and discharges of water through the Pichna River, whose estuary is located in the middle section of the reservoir. The Pichna River contains partially treated wastewater (biological treatment) from Zdunska Wola: a city with a strong clothing and textile industry as well as fruit and vegetable processing plants (http://www.zdunskawola.en). In particular, pollution from the textile and clothing industries, which use various kinds of dyes and pigments, can significantly increase the concentration of these compounds in the sediments (Gihr et al. 1991; Lexen et al. 1993; Allock and Jones 1997; Bostian et al. 2004). This may affect the increased concentration of the PCDDs/PCDFs in the sediments of the dam section of the “U” reservoir.

It should be also noted that the Pichna River flows into the reservoir at its central part, above the middle sampling point, after flowing through fish ponds. As a result, the remains of fish feed (fish meal) are deposited in the bottom of the ponds, followed by the fish feeding (Urbaniak et al. 2013). The further resuspension of such contaminated sediments due to fish foraging, and their transport together with the Pichna River waters, may increase the degree of contamination of sediment from the dam site of the “U” reservoir. A national survey of the PCDDs, PCDFs and dl-PCBs content of fish meal indicated that 95 % of the samples exceeded the prescribed limits. The TEQ concentration of these samples ranged from 1.25 to 3.66 ng TEQ kg^−1^ (Lizak et al. 2005; Piskorska-Pliszczyńska et al. 2008). Thus, feeding of farmed fish feed based on fish meal can affect water pollution and sediment in the dam section of the “U” reservoir.

Despite the total and individual concentrations of the PCDD/PCDF congeners, the obtained results showed that TEQ concentrations decreased along all three reservoirs from the middle to the dam sections by up to 52.0 % (*p* < 0.05) (Table 2). This may be connected with the reduction of the homologue groups of PCDDs/PCDFs with high TEF value, such as pentaCDD/pentaCDF (TEF = 1, TEF = 0.3, and TEF = 0.03) and hexa-CDD/hexaCDF (TEF = 0.1) along the reservoirs (Tables 2 and 4).

### A reduction of water pollution by reservoir through sedimentation process

Reservoirs, due to their higher concentration of suspended matter in comparison to river waters, are subject to a stronger influence from the sedimentation process (Kowalewska et al. 2003). The sedimentation process is faster in reservoirs with long retention times or greater amounts of suspended organic matter in flowing water. Therefore, the retention time may be one of the factors affecting the amount of matter deposited in the reservoir sediments and the micropollutants associated therewith. Consequently, the sedimentation and deposition processes may affect the reductions of PCDDs/PCDFs concentration and toxicity observed in the Sulejow Reservoir (“A”) (Fig. 2, Table 4 and 6). Greater decreases of total PCDDs/PCDFs content and TEQ concentration were observed during the high flow conditions (high water level) (Fig. 2). This may be connected with the fact that during flooding, especially in the early stages, an elevated amount of matter coming from the rapid erosion of the catchment is brought into the reservoir (Wagner and Zalewski 2000; Kiedrzyńska et al. 2008a). According to Kiedrzyńska et al. (2008b), the concentration of suspended matter in the Pilica River was 0.60 mg L^−1^ at minimum flow, but amounted to 63.3 mg L^−1^ during high water flow. In the same year, the Sulejow Reservoir, located in the middle stretch of the Pilica River, experienced a reduced SPM concentration of 45.0 %, TP of 28.0 %, and SRP of 11.0 %: when comparing reservoir inflow with outflow (Urbaniak et al. 2012b). These findings show that a reservoir may act as a system wherein water purification occurs due to sedimentation of SPM and organic matter and any pollutants associated with them.

In the present study conducted in 2010, an 81.3 % reduction in the mean annual concentrations of SPM was observed from reservoir inflow to outflow as well as 28.2 and 14.6 % decreases in mean TP and SRP concentrations, respectively. In this period, the Pilica River transported very high loads of nutrients and suspended matter to the Sulejow Reservoir due to a flood lasting for 291 days, which intensified erosion, surface runoff, and transport to the reservoir.

This may be confirmed by the spatial distributions of the PCDDs/PCDFs in the sediments, wherein the highest rate of accumulated compounds was noted in the sections of low water flow, and hence high sedimentation rate, as well as at locations where the other point pollution sources occur. Nevertheless, the high reductions of TEQ concentration, of up to 52 %, in sediments along the transect from the middle to the dam section of each reservoir suggest also that other processes, including biological and photochemical transformations, influenced the final fate of the analyzed compounds. The observed decline in the amounts of penta-CDD/CDF, hexa-CDD/CDF, and hepta-CDD/CDF congeners towards the reservoirs (Table 4) may indicate the transformation of higher chlorinated congeners containing five, six, and seven chlorine atoms to less chlorinated forms under seasonal anaerobic conditions occurring in the reservoir sediments (Nam et al. 2006; Field and Sierra-Alvarez 2008; Liu and Fennell 2008; Bunge and Lechner 2009; Urbaniak 2013).

## Summary

The obtained results demonstrate that reservoirs are effective in the accumulation of PCDDs/PCDFs in sediments due to sedimentation and deposition processes and probably their further reduction via biological and photochemical transformations. This may improve the ecological status of the river ecosystem below the dam and reduce the impact of PCDDs/PCDFs on the coastal area, which is concordant with the European Parliament and the Council Directive no. 2008/56/EC. The river itself is not such an efficient reducer of PCDDs/PCDFs, as numerous publications demonstrate an increase in their concentration along the continuum.

## Abbreviations

PCDDs: Polychlorinated dibenzo-*p*-dioxins
PCDFs: Polychlorinated dibenzofurans
TEQ: Toxic Equivelent calculated using TEF proposed by WHO in 2005
SPM: Suspended particulate matter
PIM: Particulate inorganic matter
POM: Particulate organic matter
SRP: Soluble reactive phosphorus
TP: Total phosphorus
“I”: Industrial/urban/agricultural
“U”: Urban/agricultural
“A”: Agricultural/rural
